# Is it worth it? Carers' views and expectations of residential respite for people living with dementia during and beyond the COVID‐19 pandemic

**DOI:** 10.1002/gps.5680

**Published:** 2022-01-22

**Authors:** Kritika Samsi, Laura Cole, Katharine Orellana, Jill Manthorpe

**Affiliations:** ^1^ NIHR Policy Research Unit in Health and Social Care Workforce King's College London London UK; ^2^ Geller Institute of Ageing and Memory University of West London London UK

**Keywords:** care homes, carers, Covid‐19, dementia, qualitative, respite

## Abstract

**Objectives:**

The Covid‐19 pandemic has taken a heavy toll on many people living with dementia and carers. Caring for a person living with dementia at home with limited avenues for support and a break challenged many carers. Care homes in England closed to visitors, with very few offering opportunities for a short‐stay. We investigated impact of Covid‐19 on views and expectations of carers of people living with dementia about residential respite.

**Methods/Design:**

Qualitative interviews with 35 carers were conducted March–December 2020: 30 women and 5 men, with ages ranging 30–83 years. Interviews explored experiences, views of residential respite, and expectations post‐Covid. Data were thematically analysed and salient concepts were drawn out and discussed within the research team and study advisers.

**Results:**

Three themes were identified in transcripts, relating to impact of Covid‐19 on views and expectations of respite: (1) Carers described regularly *negotiating risks and stresses of Covid*, weighing up how to prevent infection and changing family arrangements to facilitate caring; (2) Carers were *balancing different needs*, prioritising needs of their relatives while bearing the impact of cumulative caregiving responsibilities. (3) *Uncertainty about future residential respite* continued, in terms of availability, ongoing restrictions and trustworthy information sources.

**Conclusions:**

Residential respite is a positive, acceptable option for some carers to get a break from caring. Covid‐19 may have heighted some of caregiving stressors and there may be an increased need for a break. Views of care homes developed during the pandemic suggest that individual confidence to use respite may need to be rebuilt.

## Key points

1. The Covid‐19 pandemic increased demands on carers of people living with dementia with fewer opportunities for social contact and breaks
2. Carers described myriad ways to negotiate risks and stresses of Covid by developing ways to prevent infection and changing family arrangements. Carers also talked of balancing different needs, ways of prioritising the needs of their relatives while facing the impact of cumulative caregiving responsibilities
3. The uncertainty about future residential respite in terms of its availability, ongoing restrictions in care homes, and which sources of information to trust was noteworthy
4. The Covid‐19 pandemic may have heighted some demands of caregiving, and needs for a break may be greater. Individual confidence to use residential respite may need to be rebuilt

## 1 INTRODUCTION

The global Covid‐19 pandemic has taken a significant toll on many people living with dementia and their family and friends. In the United Kingdom (UK) social restrictions and closure of community services placed additional strain on dementia carers^1^, ^2^, ^3^ and carers more generally.^4^ While some healthcare and community services moved online; with varied accessibility and acceptability,^5^ personal care, socialising, and monitoring at home often continued or became entirely undertaken by family members. For those receiving domiciliary services (home care) these services were badly affected by staffing shortages and some families, fearing infection, declined such support.^3^ There is evidence that moves to care homes during the first year of the pandemic decreased with family carers stepping in to support relatives at home, for what turned out to be longer than expected by some.^6^

In England and other parts of Europe many care homes were severely affected during the pandemic by both rising rates of infections and deaths among residents and staff.^7^, ^8^ Care homes closed to visitors to limit infection; and those that offered respite care (a temporary overnight stay or day care) either stopped this option (also known as short‐break or replacement care) or offered it with numerous restrictions (such as isolating in one's own room for 2 weeks before mixing with other residents). While there is some mixed evidence of when residential respite care in a residential facility for individuals may be effective,^9^, ^10^ a temporary break of any sort provides valued support to some families and people living with dementia alike.^1^

While some service offers moved online^11^, ^12^, ^13^ there were fewer opportunities to arrange out of home breaks. In this context, we sought the views and expectations of family carers of people living with dementia about residential respite in a care home, and its post‐pandemic future. This helps to partially address Neville and colleague's^14^ encouragement of researchers to explore how and why diverse carers of people living with dementia take up (or not) different types of respite, their dis/satisfaction and their views of its outcomes; and provides new evidence of the possible implications of Covid‐19 on services for people living with dementia and their carers.

Data for this paper are from a larger 2‐year study funded by Alzheimer's Society investigating the experiences of access, use and outcomes of residential respite for older people living with dementia and family carers in England. Interviews were conducted between March 2020 and December 2020, spanning two UK national lockdowns, severe curtailment of visits to care homes, shielding of vulnerable people, and additional localised restrictions. The overarching aim for this paper was to increase understanding of the impact of Covid‐19 on the views and expectations about residential respite of carers of people living with dementia. Respite in this paper refers to ‘residential respite’ or a person living with dementia staying for a short period of time in a care home.

## 2 METHODS

### 2.1 Study approach

Using a qualitative approach, we undertook one‐off interviews via telephone and video‐call applications (apps). We offered participants the choice of video or telephone depending on which they felt comfortable with, as video interviews were still a new experience for many at the beginning of our study. The study followed principles of rigour or trustworthiness, including applying credibility and transferability (thick description of study context and individual participant characteristics), aiming for authenticity and transparency in reporting (clear paper trail), and conducting researcher reflexivity (thoughtful description of research team and considering the various strengths and limits of each member); while acknowledging the limits of these principles.^15^

### 2.2 Recruitment

We aimed to interview family carers of older people living with dementia from three categories of interest to the study: those who had experiences of residential respite, those who had declined residential respite, and those who were planning to access residential respite but had not yet taken it up. We further aimed for a diverse sample (ethnicity, gender, age, relationship type) to gain a breath of knowledge and experiences. We registered the study on the Join Dementia Research network and advertised via Twitter, as well as publicising the study through local and national voluntary groups and care home networks. Recruitment was undertaken until data saturation was achieved,^16^ or when no new trends were identified during interviews amongst each of the different subgroups of participants.

### 2.3 Data collection

All interested and eligible participants were interviewed at a mutually convenient time. We audio‐recorded all interviews with permission. Original interview topics which were pre‐pandemic focused simply on the experience of residential respite so these were re‐designed to include questions about the pandemic. The final semi‐structured interview questions that are relevant to this paper focused on capturing carers' experiences of supporting a relative or friend living with dementia during the pandemic, views of residential respite during the pandemic, and expectations of the future of residential respite. Participants' demographic information was also recorded.

### 2.4 Ethical considerations

We obtained ethical approval from King's College London Research Ethics Committee (ref. HR‐18/19‐10641) in August 2019 and sought an amendment in June 2020 for a refocus of study aims due to Covid‐19 before proceeding. We followed ethical processes and reassured participants of confidentiality and anonymity, including informing participants of their right to withdraw or terminate the interview. Processes of informed audio‐recorded consent were undertaken before proceeding with interviews. The interviewers were alert to the possibility of distress and planned to offer to stop or pause the interview should the participant appear to feel uncomfortable or similar. A safeguarding protocol was in place should we hear about or witness possible harm, and a ‘contact sheet’ of helpful resources was made available to participants.

### 2.5 Data analysis

Interview audio recordings were transcribed verbatim, and first transcripts were analysed using principles of thematic analysis.^17^ Inductive analysis focused on identifying key trends or broad themes. Initial line‐by‐line coding was conducted on a first randomly selected set of participant transcripts. A broad coding framework focusing on descriptive themes was developed from this analysis which was then applied to all transcripts. When all data were coded at a descriptive level, key themes were discussed within the study team and higher order interpretations were applied to the coding framework. Analytical discussions with the rest of the study team enabled different perspectives and assumptions to be challenged. The process of analysis was clearly documented in a rigorous paper trail via notes and memos to ensure authenticity and demonstrate rigour. The research team was female, with backgrounds in gerontology, health and care research, family caregiving, and care home governance, each with over 10 years' experience in dementia and social care research. While this aided the recruitment and data collection parts of the study, we were mindful during analysis to bracket our experiences and knowledge of extant literature and remain true to participant accounts. Emerging findings were presented to the study advisory group (comprising care home providers, social care and dementia experts, and people affected by dementia) in an online meeting for their reflections on how they accorded with their pre‐Covid experiences. We have adhered to the Standards for Reporting Qualitative Research^18^ to demonstrate transparency, authenticity and credibility.

## 3 PARTICIPANT CHARACTERISTICS

We conducted 34 interviews with 35 carers (2 carers were jointly caring for a relative). Four were former carers who shared their views about residential respite (for 2 their relative had moved pre‐Covid to a care home and for the other 2 their relative had recently died). There were 30 women and 5 men, age range 30–83 years. Thirty were White British. There were equal numbers of adult children and spouse/partner carers. All but two reported being heterosexual, and 29 participants lived in owner‐occupied homes, the majority housing tenure in England (see Table 1). All interviews were conducted in English and all participants lived in England.

**TABLE 1:** Characteristics of carers interviewed (34 interviews, 35 participants)

	Total
Gender	
Female	30
Male	5
Age range (in years)	30–83
0–29	0
30–39	1
40–49	2
50–59	10
60–69	11
70–79	9
80–89	2
Ethnicity	
White	30
Asian	4
Indian	2
British	2
Black	1
Caribbean	1
Relationship	
Spouse/partner	15
Parent	15
Sibling	1
Other family member	4
Sexuality	
Heterosexual	33
Gay/lesbian	1
Bisexual	1
Housing type	
Owner occupied	29
Rented privately	2
Rented Local Authority/Housing Association	4
Religion or belief	
Christianity	21
Sikhism	1
Islam	1
Hinduism	1
Buddhism	1
Other	4
No religion or belief	6

*Note*: Two carers, caring for the same person, participated in one interview.

## 4 FINDINGS

Three salient themes identified in transcripts related to views and expectations of residential respite were:

(1) Negotiating the risks and stresses of Covid‐19,
(2) Balancing different needs,
(3) Continued uncertainty about future respite services and future support in a post‐Covid world.

Each theme and associated sub‐themes are described more fully below, with a participant quote to typify the theme (Table 2). No within‐ or between‐group differences were noted amongst participants who had accepted, declined, or were awaiting respite.

**TABLE 2:** Themes and sub‐themes

Theme	Sub‐theme
(1) Negotiating the risks and stresses of Covid	(1a) Preventing infection
	(1b) Changed family arrangements
(2) Balancing different needs	(2a) Prioritising the needs of their relative
	(2b) Different carer “breaking points”
	(2c) Impact of cumulative caregiving responsibilities
(3) Continued uncertainty about future respite and future support in a post‐Covid world	(3a) Availability of residential respite
	(3b) Worry about ongoing restrictions in care homes
	(3c) Information sources

### 4.1 Negotiating the risks and stresses of Covid‐19

#### 4.1.1 Preventing infection

Participants described several lengths they went to prevent the spread of Covid‐19 infection. Usual sources of social contact and daily respite or breaks (such as day centres and church groups) closed at the start of the UK government's first national lockdown on 23rd March 2020. However, many carers reported that they avoided shops, postponed non‐urgent medical appointments, and cancelled homecare workers in order to cease external social contact and its risks of infection. One participant explained how she tried to keep her husband safe, and why any type of care outside of the home (including residential respite) was not an option she considered:

The worry of it… [my husband] is very poorly, and I didn't want him even going to the hospital. I just feel that if you're out of your own little bubble, you become more vulnerable. If he was out of his little bubble, I think he'd be far more susceptible you know to get the Covid, and I don't think he would physically cope with it cause he's got lung problems anyway. Respite, day centres, anything like that… even hospital appointments we have done on the phone… He can go in my car and if I take him somewhere, then I will know 99.9% we're okay. (Carer 06, declined respite).

Another carer described a friend's experience of trying to prevent infection resulting in breaking point when trying to manage alone that necessitated moving her father to a care home:

A month after [lockdown], [her father] went further and further downhill and really, she just couldn't cope. So he got admitted [to a care home] and within a few weeks, he died of Covid… I'm so upset. I'm really, really angry, especially because my friend's father has died and I believe that it’s due to lack of PPE (personal protective equipment); I really do. The irony of this thing is that [my friend] was afraid for the home carers to come into help so she stopped the carers from coming. And we are Afro Caribbean background, the ethnicity also they started to say play a part that we are more susceptible to Covid‐19 and stuff like that. So [my friend] understood those things and, so that's also playing on her mind so she stopped the carers. So she took [father's personal care] on and it all just broke down (Carer 14, had experience of respite).

#### 4.1.2 Changed family arrangements

Several carers described receiving additional family support, such as when adult children worked as a team around the family to prevent infection, such as from homecare workers. Some had provided a chain of relay‐style support, but this was a temporary arrangement; a couple of carers had made arrangements such as hiring live‐in carers to provide them with a break at home. Most were adult children supporting older parents, involved in either shopping, delivery or picking up prescriptions, or a doorstep ‘checking’ visit rather than personal care. In two families, these arrangements overlapped with providing childcare when schools closed. Such participants felt their employers were sympathetic to these circumstances and noted how working from home made this feasible:

[My daughter] stayed here for a month and all of the girls' employers are aware of our situation. They're very supportive. But at that stage everybody was working from home anyway and they were all told, ‘No, you can go and support your family and work from home’ in the knowledge that the work from home would be not as active as normally (Carer 09, declined respite)

### 4.2 Balancing different needs

#### 4.2.1 Prioritising the needs of their relative above their own need for a break

Participants described subjugating their own often increased needs for support with what they deemed was in their relative's best interests. While some were able to stop homecare services and enlist family help, others sacrificed their breaks. One carer had put aside plans since she was unwilling to use residential respite care while rates of Covid were rising:

Well, I got to the stage where I'd got about half a dozen or so care homes [for residential respite] and I'd mentioned it to social services, to the social worker, that this is what I was planning to do, and she was also going to come back to me and give me a list of where she thought that there might be space available at that particular time. So I sort of got sort of three quarters of the way there [laughs]… I identified the time, I'd got accommodation going to stay with my friend. I'd got that all set up and this was just the final hurdle was which home was he going to go to? And then we were going to face the ‘telling him he's going in stage’… [laughs]… It was finally getting there. You know, I was looking forward to seeing, I mean, friends that I haven't seen there for three years. And it was all in place and we talked about exhibitions we're going to see in [town] (Carer 03, awaiting planned respite).

Two other carers had put residential respite plans on hold when the first UK lockdown started but hoped to resume these when possible. One was deferring a residential respite break as she feared her husband would be confined to quarantine in his bedroom for the first week of his stay. Such isolation would be unacceptable to her and other carers she knew:

I've more or less accepted the fact that the respite is certainly not going to happen for a while, because as I said to you, there's no way I would expect [husband] to go in somewhere and spend a week in his room… There won't be any respite for me or for any of our friends' group, certainly not (Carer 02, awaiting planned respite).

#### 4.2.2 Different carer “breaking points”

There were reports of additional and increased challenges when caring for someone living with dementia at home during the pandemic. These included social networks contracting, independence declining, and cognitive impairment increasing in their relative. Carers variously described feeling isolated, burnt out, and tired, as several of their usual channels for face‐to‐face social contact and getting a break, meeting family and friends, were no longer available. The term ‘burnout’ was given as the reason why a break would be necessary once things felt safer and several participants acknowledged that they badly needed the physical and mental break of residential respite. However, carers recounted different experiences according to their feelings about managing their relatives' symptoms, and different near “breaking points” were described:

Participant: *It's really going to depend on how I get on with my husband as to whether I feel I get to the point where I really, really, it'd be detrimental to us for me to keep him here [at home]*.
Interviewer: And how have you been coping over the time with Covid and the lockdown that we had?
Participant: *About the same as everyone else I think. You have good days, you have bad days* (Carer 01, awaiting planned respite).

#### 4.2.3 Impact of cumulative caregiving responsibilities

Intertwined with the individuality of carer “breaking point,” was the recognition from some participants that cumulative and other non‐caring stressors could newly lead to deciding upon residential respite or permanent care home move:

My brothers are quite concerned that my sister and I kind of have a bit of a burn out and other things are impinging on our care responsibilities to be honest with you, so that's why the discussion is there… this was pre‐Covid but even more since Covid, because my sister and I have now taken on more of the care responsibilities so that might become an issue as the winter comes on you see. So, yes, we have had a discussion and also had the opportunity to visit a couple of people in a couple of care homes locally (Carer 07, declined respite).

### 4.3 Continued uncertainty about future residential respite and future support in a post‐Covid‐19 world

#### 4.3.1 Availability of residential respite

Participants highlighted concerns about local residential respite provision in a post‐Covid world. Some had seen local or preferred care homes closing permanently during the pandemic. One reported difficulty finding another care home for respite:

We were hoping to go back [to care home for residential respite] this year, but of course what with Covid, that stopped. And [care home] has in actual fact gone bust, so there won't be that opportunity to do that again and that was the only place that I found that sort of catered for us. [Manager] had sort of got quite a lot of links in the local community, places to visit… So, there was a lot of caring and thought gone into those sorts of things. And it's closed. I did ring [manager] and she was just saying obviously business was finished and she just felt like if she'd been younger she said she would've probably tried to keep going. ‘Well’ she said, ‘I'm 60 now and I just don't feel I've got the time to have the business recover from all of this' and it was a shame, it was such a shame (Carer 12, had experience of respite).

Another carer talked of local care homes currently only accepting emergency cases for residential respite and hoped this would be temporary:

We had a fair few care homes [around here] where they had a lot of residents die very quickly over a short length of time. All the care homes have been through deep cleans and all sorts. Some are still not accepting new people, so there is not the availability but, actually, if it's an emergency you're more likely to be. So, I think at the moment, probably for the next six months, it would have to be emergency use for respite only rather than planned respite (Carer 05, had experience of respite).

Two carers described the worries of finding regular residential respite because care homes were not currently taking advance bookings. Many remarked that existing lack of options had been exacerbated since the pandemic:

And so now I've got to hunt round, and it's going to be really, really difficult to find respite care, the care that this care home had filled up and they said, “we're not going to do bookings as far ahead as we used to”. Because when he came out [of residential respite after] one week, I could book one in four months' time, they said “no you're not going to be able to do that now; maximum forward booking would be a month” so everything becomes precarious. And now I've got to hunt and find another place; but I'm hoping, with the help of the Admiral nurse (community‐based dementia nurse), to find somewhere, but I don't know whether there's anywhere locally taking people for respite care given the Covid situation. (Carer 06, had experience of respite).

#### 4.3.2 Worry about ongoing restrictions in care homes

Interviews highlighted carers' worries and anxiety about lingering Covid‐19 restrictions in care homes that they had heard about from friends, local networks, local social media and local newspapers. For instance, being quarantined before interacting with other residents, along with encountering staff in full personal and protective equipment (PPE), were viewed as deterrents for anyone considering respite during the pandemic, and no‐one knew how long these restrictions would remain:

I would be concerned if he went in [to a care home] now and he was isolated in a room, you know, and everybody that went in was gowned up, masked up, you know, and took him his food in and all he could do was sit in a chair, alright he would have a television but sit in a chair and have no interaction with anybody because he can't shut up. He likes to talk [laughs]. [And just thinking…] Where would he stay? And who would he see? And would he be allowed out at mealtimes or would he be allowed out so he could wander the grounds, or wander… I'm thinking yeah, you know could he just wander himself out there? Or would somebody have to go with him? (Carer 11, declined respite).

The prohibition of care home visiting due to Covid‐19 restrictions had also created new fears about any possible move to a care home as the future was so uncertain.

#### 4.3.3 Information sources

We asked participants about their sources of information during Covid‐19, to situate the contexts through which carers were accessing and receiving information and advice about residential respite. Many reported accessing both national and local television news. Nearly all watched the daily government briefings on BBC television during the first lockdown. Some however said they began to find these too disheartening and, as time went on, viewed the evening news updates rather than watch live briefings. Facebook and other social media were also relied on for news by those with online access. One carer regularly followed the local newspaper's updates via social media for information about local care homes and community resources. Such information was informed by community contact:

I've been following in the local newspapers what the death rates have been in the area and which death rates have been in the area from care homes. And also I've got people who, I mean there's a care home on the estate where we live and I've heard from people who live in the sheltered accommodation about the number of deaths that have been there. And there's another care home nearby and I'd heard about the fact that there'd been a lot of deaths there and actually it's been so bad that the manager had been sacked (Carer 03, awaiting planned respite).

Notably few reported professionals discussing local care markets with any precision about what would likely be coming available.

## 5 DISCUSSION

Carer stress when supporting people living with dementia has long been recognised^19^ and several options have been developed to ameliorate this, including respite at home and in residential facilities. Willingness to take a break in the form of residential respite however will still depend on individual confidence, information, family support and not least the recovery of the care home sector so that it can meet demand and develop trusted provision. As Phillipson and colleagues observed,^20^ we need to move beyond simple categorising of carers as ‘users’ or ‘non‐users’ of respite, and this may be particularly so in the pandemic context.

Like others reporting carers' experiences^21^ and for dementia carers,^5^ we found some cancelling of homecare services and minimising of social contact during the pandemic which offered security yet compounded isolation. Additional family support was welcomed when it occurred, but may have suppressed need and been temporary.^22^ Fears of future pandemics will be interesting to assess in research exploring the acceptability of residential respite care and its addition to the factors influencing take‐up.

Maintaining the option of residential respite is uncertain when vagaries of supply and demand are inter‐related. On the demand side, worries about care homes' ongoing restrictions may well endure, and information needs to be clear about the implications of infection control measures for temporary residents and their relatives. Respite is an acceptable option for some carers but needs to be acceptable in terms of care culture and socialisation, or ‘collaborative solution‐focused care culture’ as suggested by O’Shea and colleagues.^23^ Dementia care professionals could be accurate sources of information as well as counselling carers about wider options, considering their own needs and wellbeing, and problem framing. Some carers may have found the pandemic has further reinforced their antipathy to any residential respite care,^24^ and professionals will need to be skilled in supporting people to take a break that may be home based. Unlike accounts of day services providing respite whose staff were sometimes redeployed to contact previous attenders,^3^ we heard no reports from our interview data of residential respite services contacting regular clients, although the severe pressures on care homes may well account for this.

On the supply side, care homes' engagement with the wider community such as open days and activities, the encouragement of visitors and volunteers^25^ may need to be further supplemented by mainstream and social media publicity illustrative of respite as a positive option. Our study indicates the reliance of carers on multiple sources of information from local sources and that many are aware of their local care market's reputation and staffing. Putting a trusted face to the name of a service may assist in developing a sense that individuality of the person living with dementia and the carer will be acknowledged.

Our participants were mainly from relatively affluent groups as judged by high levels of owner occupation – although this is the majority housing tenure in England^26^ – and regular internet use. Residential respite is a means‐tested service in England, and carers have elsewhere mentioned that costs may be prohibitive when self‐funding.^4^ This limits the generalisability of this study although we had a broad sample group in terms of respite use, gender, relationships, age, religious and ethnic diversity. Many of the problems reported by our participants were also mentioned in the Alzheimer's Society's^1^ survey of nearly a thousand carers. Our interviews spanned several months, starting in March 2020 when the pandemic was fully acknowledged in the UK, and covering periods of stronger and weaker restrictions up to December 2020; we did not find substantial differences however in carers' reports of respite access other than an impression of growing tiredness. This reflects the continued restrictions on care homes unlike homecare services which seem to have been curtailed initially but then resumed for older people.^27^

### 5.1 Limitations

This paper is limited by focusing only on the views of carers and not their relatives living with dementia who had few opportunities for social interaction or a break during the pandemic too. The sample was predominantly White British and female, despite efforts to recruit a more diverse sample. Findings therefore do not capture some of the perspectives which may have been unique to specific groups, such as fears of Covid‐19 amongst black and minority ethnic groups. Most participants owned their own homes, which may have had a bearing on their ability to pay for their own residential respite place. The strengths of the study lie in there being an equal mix of spouse carers and adult children, and the interviews being conducted at a unique contextual time.

## CONFLICT OF INTEREST

The authors have no conflicts of interest.

## Data Availability

The data that support the findings of this study are available from the corresponding author upon reasonable request.
